# Societal Impacts of Pandemics: Comparing COVID-19 With History to Focus Our Response

**DOI:** 10.3389/fpubh.2021.630449

**Published:** 2021-04-12

**Authors:** Grace E. Patterson, K. Marie McIntyre, Helen E. Clough, Jonathan Rushton

**Affiliations:** Department of Livestock and One Health, Institute of Infection, Veterinary and Ecological Sciences, University of Liverpool, Liverpool, United Kingdom

**Keywords:** plague, smallpox, Spanish flu, economic impact, pandemic control, COVID-19

## Abstract

COVID-19 has disrupted everyday life worldwide and is the first disease event since the 1918 H1N1 Spanish influenza (flu) pandemic to demand an urgent global healthcare response. There has been much debate on whether the damage of COVID-19 is due predominantly to the pathogen itself or our response to it. We compare SARS-CoV-2 against three other major pandemics (1347 Black Death, 1520's new world smallpox outbreaks, and 1918 Spanish Flu pandemic) over the course of 700 years to unearth similarities and differences in pathogen, social and medical context, human response and behavior, and long-term social and economic impact that should be used to shape COVID-19 decision-making. We conclude that <100 years ago, pandemic disease events were still largely uncontrolled and unexplained. The extensive damage wreaked by historical pandemics on health, economy, and society was a function of pathogen characteristics and lack of public health resources. Though there remain many similarities in patterns of disease spread and response from 1300 onwards, the major risks posed by COVID-19 arise not from the pathogen, but from indirect effects of control measures on health and core societal activities. Our understanding of the epidemiology and effective treatment of this virus has rapidly improved and attention is shifting toward the identification of long-term control strategies that balance consideration of health in at risk populations, societal behavior, and economic impact. Policymakers should use lessons from previous pandemics to develop appropriate risk assessments and control plans for now-endemic COVID-19, and for future pandemics.

## Introduction

COVID-19 has disrupted everyday life worldwide. It is the first disease event since the 1918–20 H1N1 Spanish influenza (flu) pandemic to demand an urgent global healthcare response, propagated by the speed and likelihood of potential transmission. An understanding of how much disruption is caused by the pathogen, and how much is caused by our reaction to its potential presence, is essential. We compare SARS-CoV-2 against three other pathogens known for the magnitude of their impact. *Yersinia pestis*, causative agent of the 1347 Black Death, is among the most destructive pathogens in human history. Variola major, cause of the 1520s smallpox outbreaks in the New World, exemplifies how disease impacts vary by population. Spanish flu is most similar to the current pathogen, yet major differences exist regarding scientific advancements and pre-existing immunity.

We compare across these four major disease events the rates of infection, likelihood of dying, and available diagnostics, therapeutics and vaccines (Table 1). We examine the historical impact of these largely unchecked pathogens upon populations and economies. We discuss how culture and society's collective memory affect the response to pandemics and identify important lessons for decision-making as we adapt to a new normal.

**Table 1:** Context and impacts of major pandemics.

	**Black Death 1347–1351**	**Smallpox (New World) 1520–1527**	**Spanish Flu 1918–1920**	COVID-19 2020^*^
World Population (1)	364.8 million	450.8 million (Americas 60.5 million) (2)	1.86 billion	7.80 billion
Mortality (% global population)	In Europe, ≥25 million (25–75% of European population) (3)	2–15 million Aztec deaths (4) 200,000 Incan deaths Major contributor to 90% population decline in the Americans from 1,500 to 1,600 (2)	17.4–50 million (1–3%) (5, 6)	1,664,344 (0.02%) (7)
Case Fatality Rates	Bubonic plague: 50–60% Pneumonic or septicemic plague: approaching 100% (8)	Estimated ≥50% among Aztecs and North Americans (9)	2–3% (5)	0.25–3.0% (10) (lower estimates more likely)
Number Infected	Unknown	Unknown	500 million (clinically apparent) (5)	75.1 million (7)
Notable Risk Factor(s) for Severe Disease	Overcrowding, poor housing; proximity to fleas and animal reservoir (8)	No previous exposure to disease in region (“virgin soil”) (4)	Healthy 15–40 year olds, secondary bacterial infection (5)	Old age, pre-existing conditions (11)
Transmission	Flea bite or close contact with respiratory droplets of a pneumonic plague patient (8) Low direct interpersonal transmission	Contact with respiratory droplets or aerosols. Patterns of behavior (contact with the sick) likely enhanced spread (12) Low dose can be infectious (13)	Contact with respiratory droplets or aerosols (5)	Contact with respiratory droplets or aerosols (14).
Available medical interventions and scientific understanding	No knowledge of germ theory; contemporary physicians admitted no known effective cures or treatments and recommended fumigation, bleeding, purging, etc. (15)	No knowledge of germ theory or effective treatment among Europeans or Americans. Among Native Americans, isolation and traditional medical practices (e.g., sweat lodges, bathing) (9)	Knowledge of germ theory but misidentification of aetioglocal agent. Palliative care and homeopathy employed. No vaccine, antiviral, ventilators, or antibiotics for secondary pneumonia (16)	Causative virus isolated and genome sequenced. Vaccines in advanced trials and roll-out, general antiviral, anti-inflammatory, and antibiotic medications available. Ventilators and modern medical practice in use (14)
Disease Control Strategy (at the time)	Travel restrictions and isolation at the city-state level. Usually severely enforced and aimed against specific people groups (17) First record of quarantine.	Minimal; among Native Americans, little evidence for isolation of the sick or other nonpharmaceutical interventions (12)	Masks, social distancing, public closures, limits on public gatherings. Poorly and sporadically enforced; ineffectual and too late (17)	Near-global lockdown, quarantine, masks, track and trace (14) Implementation varies by country, ranging from highly successful to poorly enforced.
Population Effects	Strong, lasting effect negative effect on global population growth. European population did not recover to pre-plague levels until mid-16th century (18)	Minimal effect on global population growth, but wiped out populations in New World (~90%), extinction of some people groups (4)	Temporary global population growth decline during period of outbreak (6)	No anticipated effects on global population growth. Corollary effects on public health may reduce life expectancy (19)
Long Term Economic Effects	Labor shortages led to higher wages, European peasant revolts, and shifts in sociodemographic power dynamics. Increased innovation and mobility of labor (18)	Shift balance of power in New World, leading to societal collapse of Native Americans and enriching colonial European powers (4)	Limited and obscured by WWI. Sharp but short-term effects on industry. Entry of new groups into labor force (20) Towns that had quick shutdowns fared better	Unclear; driven by large-scale response methods and spontaneous reduction of economic activity. Predicted surge in poverty, lower investment, reduced global trade. Strongest impact in developing economies (21)

* *Figures accurate as of 18 December, 2020*.

## Epidemiology

SARS-CoV-2 differs from *Y. pestis*, V. major and Spanish flu in terms of disease transmission and pathophysiology. Of these four, it is the least deadly, and poses the lowest risk to otherwise healthy people; however increasing evidence suggests significant long-term sequelae for a proportion of individuals who have symptoms. The Black Death had exceedingly high case fatality rates (CFRs), approaching 100% for septicemic and pneumonic plague and 50–60% for the bubonic form of the disease (8). Over a third of the European population died during the 1347 outbreak, with some regions experiencing up to 75% mortality (22). CFRs for smallpox amongst immunologically naïve native Americans in the 1520s were estimated at 50% (9), and many survivors were left disfigured or blinded. Smallpox (and other European diseases) drove an estimated 90% decrease in indigenous populations in the Americas from 1500 to 1600 (2). Spanish flu had a CFR estimated at 2–3% (23) and few known long-term effects, other than occasional extended convalescence and limited instances of neuropsychiatric disorders (24). Current COVID-19 CFR estimations range from 0.3 to 3.0%, with lower estimates more likely to be accurate (10). There are growing reports of secondary and long-term impacts from COVID-19, typically among hospitalized patients but also among less severe cases. These include poor cardiovascular functioning (25), wide-ranging neurological symptoms (26), chronic fatigue (27), and others, with some patients needing long-term convalescence. While it is too early to fully understand the long-term impacts of COVID-19, similar post-viral syndromes have also been observed among those infected with SARS (28).

It is unknown how many people were infected during the Black Death and 1520s smallpox outbreaks. An estimated 500 million people (1 in 3 worldwide) were infected with Spanish flu and 1–3% of the global population died from the disease (5). Thus far, 75.1 million people have been confirmed to have COVID-19 (~1 in 104), killing 0.02% of the global population (7).

Disease susceptibility and immunological naivety influence the outcome of pandemic disease events. Both plague and smallpox are highly infectious and affect people of all ages, though smallpox exhibits a significantly higher mortality rate amongst children compared to adults (9). Spanish flu had severe impacts amongst the otherwise healthy 15–40 age group while also affecting typically high-risk groups (23). COVID-19 is different; it has a low attack rate (29) and severe clinical disease occurs mainly in the old and those with pre-existing health conditions (11).

*Y. pestis* has evolved over centuries to evade and modulate innate and adaptive immune responses (30). In 1347, naïve Europeans would have had minimal immunological protection from the plague. Conversely, pre-existing herd immunity from years of smallpox circulation spared European colonizers the widespread mortality observed among naïve populations when smallpox was introduced to the New Word (9). While Spanish flu was likely a result of a novel variant, there is evidence of cross-protection in elderly populations who were exposed to historical flu outbreaks; this was also observed among survivors of later flu epidemics (23). It is unknown if exposure to commonly circulating coronaviruses provides protection against COVID-19, but reactivity against SARS-CoV-2 has been observed in T-cells from unexposed people (31).

While much of the modern world would be unrecognizable to our ancestors, certain dynamics of disease spread remain the same. Humans and domesticated animals historically lived at close quarters, and the risk of animal to human disease transmission was intuitively minimized thousands of years before a causal relationship was established (32). Communicable diseases spread more easily where there is poverty and/or high population density (33, 34), as seen in Marseille where ~80% of the population perished in the Black Death (3). In India, Spanish flu mortality rates among members of the lowest social class were three times higher than that of other demographic groups (35). These risk factors remain relevant today: 73% of emerging infectious diseases in humans originate in animals (36), including COVID-19 (37). Large cities with international travel hubs, such as New York and London, were initially hit hard by COVID-19 and contributed to the unprecedented speed of global disease spread. Early understanding of the complex, multi-factorial role of socio-economic deprivation in COVID-19 spread, indicates that poverty remains a risk factor for poor outcome from infectious disease (38).

## Mitigation and Economic Effects

Science and public health advances have accelerated over the last 100 years; we should be better equipped to respond to the current pandemic. The Black Death, New World smallpox outbreaks, and Spanish flu all occurred before the discovery of antibiotics and antivirals and the development of centralized public health surveillance; even the aetiological agent of each outbreak was unidentified at the time. Early forms of quarantine and isolation were employed during the Black Death, and sanitary cordons were enforced by armed guards (17). Outbreak spread was ultimately unmitigated for both the Black Death and New World smallpox, and no effective treatment protocols were available (9, 39). The Native American custom of sleeping in close proximity to sick individuals would have spread smallpox even more efficiently (12). Mitigation tactics only slightly improved for Spanish flu, with sporadic use of non-pharmaceutical interventions such as track and trace, isolation, and social distancing (17). Late implementation, poor record-keeping, lack of a centralized global health body, and wartime priorities rendered these largely ineffectual. Public gathering spaces and schools were commonly shut down, but total lockdowns were not employed. Masks and disinfectants were used liberally, but ineffectively, and the only treatment was palliative care. Today, healthcare professionals can deploy antivirals, immune modulating drugs, antibiotics, oxygen, and ventilators to treat COVID-19 and related complications. At the time of writing (December 2020), the first doses of multiple vaccines for COVID-19 are being administered and surveillance systems have been established in many countries. Extensive lockdowns were enacted in most countries and travel restrictions, social distancing, and quarantine rules remain in place for the foreseeable future. Concern that healthcare capacity could be overwhelmed has stimulated rapid capacity building and shifting existing capacity away from day-to-day needs to help alleviate COVID-19. These modern tactics minimized harm from various infectious diseases but halted critical preventive activities, which may cause future chronic health burdens and global social and economic disruption surmounting that of COVID-19 alone (40). Countries such as Taiwan, which were able to locally eradicate the virus via swift but relatively short-lived enactment of nonpharmaceutical interventions, have suffered the least in terms of health, social and economic damage from COVID (41). Countries that have not been as successful in controlling spread of the virus (e.g., the United States - US) face long term health and economic damage from poorly coordinated and implemented control plans.

Historically, severity of disease has correlated with severity of economic outcomes. The Black Death caused a major labor shortage, providing unprecedented market power to common people and sparking a European peasant revolt (18). While trade and industry were temporarily damaged, the socio-economic structure of society was permanently redressed as wages increased. Skilled workers were increasingly mobile and spread innovative technology faster and further than before (18). Smallpox had less dramatic effects on the evolution of economic systems, but its unequal impacts on native groups paved the way for European conquest of the New World, through which mining of natural resources funded European empire-building (4). Smallpox often preceded the conquistadors, decimating populations and leading to starvation among survivors as their societal structure collapsed (9). Spanish flu closely followed World War I (WWI); both were particularly deadly for young to middle-aged men, which led to labor shortages and stalling of industry (20). These shortages were not as economically transformative as for the Black Death, perhaps as industry was less dependent upon mass labor, a smaller proportion of the overall workforce died, and more women and minors went into work outside the home (42). There is little evidence that Spanish flu caused major GDP or consumption declines or stock market volatility; major fluctuations had already occurred due to WWI (43, 44). These outbreaks contrast with COVID-19, which poses minimal physical risk to most of the labor force but major economic risk from the unprecedented lockdowns and non-pharmaceutical interventions employed to contain the virus. Early transient labor shortages were driven by shifts in demand and movement restrictions (45). Now, mitigation measures drive record unemployment. COVID-19 related stock market volatility is unprecedented (43) and national GDPs have plummeted (46). It remains to be seen what detrimental effects will persist in the global economy, though experts predict wage contraction and widespread poverty, with profound effects on emerging markets and developing economies (21).

## Collective Social Memory and Human Behavior

The Black Death, smallpox, and Spanish flu no longer pose an imminent threat to the global population, but they changed global population structure and economies and prompted scientific advances in disease eradication, antibiotics, vaccines, and surveillance systems. It is too early to understand the long-term effects of SARS-CoV-2 or whether we will eradicate this pathogen, but we should seek inspiration from the past for how to move forward in control.

Bubonic plague, smallpox, and Spanish flu have been controlled by herd immunity and scientific advancements, though plague and flu still circulate. Localized hotspots of infection may be our COVID-19 future as this disease becomes endemic. Over the past 80 years, significant resources were spent developing surveillance systems, vaccines, and programs to monitor and manage flu (17). For COVID-19, it is unlikely we will develop curative treatments, and, as asymptomatic cases make up an estimated 17.9–30.8% of infections, disease eradication is unlikely (47). The best approach may be that birthed from the Spanish flu: develop vaccines, efficient monitoring systems, and an understanding the epidemiology of the virus, when endemic. The “end goal” would be high-level vaccine coverage coupled with notifiable disease status. This will potentially take a long time: until this is achieved, how can COVID-19 be managed with maximal public cooperation coupled with maximizing economic activity?

Public responses to pandemic disease are largely unchanged since the Black Death. Disbelief of disease presence, misinformation, unclear public communication, disregard for governmental proclamations, and poor personal risk assessment were and are still common. Despite the rapid onset of bubonic plague, it often took weeks for plague infection to be recognized in a population. In 1630s Italy, physicians were “insulted on the streets” for warning people about the arrival of the bubonic plague (48). Today, media touting COVID-19 conspiracy theories are amplified by prominent voices (49). Conflicting information about ongoing disease has long been spread (purposely or not) by news media, sometimes at the behest of governmental leadership. In an example of wartime censorship, the Italian government forced a Milan newspaper to stop printing daily death tolls during the Spanish flu because it was too demoralizing (17). In the US, public health officials hid the extent of disease spread and downplayed the danger it posed (20). In attempts to keep morale up, leaders inadvertently eroded trust in public institutions.

Uncertainty and desperation can drive people to use of dubious modes of protection during disease outbreaks. Physicians in the 1300s recommended bloodletting and drinking wine to ward off the plague (15). During the Spanish flu people wore camphor bags and gargled saltwater, while early in the COVID-19 pandemic, many sought protection from zinc lozenges and off-label medications (50). In a parallel to modern times, official Anti-Mask Leagues were formed in the US during the Spanish flu, citing insufficient scientific evidence for mask use and violation of constitutional rights. These examples demonstrate that public response to pandemics is driven by personal assessment of risks as shaped by individual circumstances and belief systems, not necessarily government mandates. In an attempt to save their economy during COVID-19, the Swedish government did not impose lockdown. However, Sweden still experienced economic losses similar to their neighbors, as people spontaneously reduced mobility and economic activity (51), being unconvinced by the herd immunity strategy (52), and presumably having made a decision based their individual assessment of risk.

COVID-19 poses a more targeted threat to health than previous pandemics however we have more understanding of its etiology and epidemiology than would have been possible in previous centuries. Why then has our global response been so profound? Our collective understanding of pandemics, as shaped by literature and culture, may play some role. The historical fascination with plagues is evidenced by some of the earliest surviving English literature and is observed across art and entertainment. Geoffrey Chaucer's 1386 “Canterbury Tales” describes the effects of total social upheaval that arose from the Black Death and provides insight into a world shaped by the threat of plague. Albert Camus's 1947 “The Plague” accurately captures the now familiar atmosphere of lockdown, obsession with case counts, and feelings of powerlessness. More recent movies such as Outbreak and Contagion may be a modern individual's reference point for predicting the possibilities of horrific disease outbreaks (and indeed, sales of these increased markedly at the pandemic's outset) (53). All explore the effects of pandemics on fear as well as fear on pandemics (54). A specific challenge for the modern era comes via the immediacy of social media, where genuine and “fake” information are frequently presented with apparently equal credibility. The myriad collective experiences and cognitive biases innate to humanity are further challenges that scientists, policymakers, figureheads and communicators should be aware of in themselves and their audiences when formulating and communicating response plans (55).

## Conclusion

In the era of COVID-19, scientific and medical advances have enabled us to identify and treat disease in a way that would have been unimaginable to previous generations. Therefore, the biggest danger we face are reactions that are disproportionate to the nature of risks from COVID-19, leading to challenges in core social activities of food production, provision of education, healthcare, and basic health needs. Indeed, one legacy of COVID-19 may be the corollary deaths that stem from disease control strategies (40). Major economic downturns are correlated with chronic disease and mental disorder-associated mortalities; already in 2020 we have observed short-term excess deaths not attributed to COVID-19 and reduced healthcare uptake. To minimize long-term harm to global health targets, decision-makers must balance the direct health risks from the virus against those from the socioeconomic effects of control strategies. The underpinning evidence and reasoning must be unified across government, medicine, and media, and presented to a mistrustful public with transparency. As seen in the past, illogical decision-making and poor leadership have the potential to multiply harm caused by disease. We must minimize the impact of this pandemic by accurately assessing and proportionately responding to the true threats of COVID-19 and its legacy.

## Data Availability Statement

The original contributions presented in the study are included in the article/supplementary material, further inquiries can be directed to the corresponding author/s.

## Author Contributions

The idea for this paper was initiated by JR and developed by GP, with input from KM and HC. GP wrote the first draft, with edits by JR, KM, and HC. All authors contributed to the article and approved the submitted version.

## Conflict of Interest

The authors declare that the research was conducted in the absence of any commercial or financial relationships that could be construed as a potential conflict of interest.
